# New Perspectives on Lung Cancer Screening and Artificial Intelligence

**DOI:** 10.3390/life15030498

**Published:** 2025-03-19

**Authors:** Leonardo Duranti, Luca Tavecchio, Luigi Rolli, Piergiorgio Solli

**Affiliations:** Thoracic Surgery Unit, Fondazione IRCCS Istituto Nazionale Tumori, 20131 Milan, Italy; luca.tavecchio@istitutotumori.mi.it (L.T.); luigi.rolli@istitutotumori.mi.it (L.R.); piergiorgio.solli@istitutotumori.mi.it (P.S.)

**Keywords:** AI in lung cancer screening, artificial intelligence in CT imaging, biomarker-driven screening for lung cancer, liquid biopsy in lung cancer detection, artificial intelligence in PET imaging, screening, lung cancer, artificial intelligence, low-dose CT scan

## Abstract

Lung cancer is the leading cause of cancer-related death worldwide, with 1.8 million deaths annually. Early detection is vital for improving patient outcomes; however, survival rates remain low due to late-stage diagnoses. Accumulating data supports the idea that screening methods are useful for improving early diagnosis in high-risk patients. However, several barriers limit the application of lung cancer screening in real-world settings. The widespread diffusion of artificial intelligence (AI), radiomics, and machine learning has dramatically changed the current diagnostic landscape. This review explores the potential of AI and biomarker-driven methods, particularly liquid biopsy, in enhancing early lung cancer detection. We report the findings of major randomized controlled trials, cohort studies, and research on AI algorithms that use multi-modal imaging (e.g., CT and PET scans) and liquid biopsy to identify early molecular alterations. AI algorithms enhance diagnostic accuracy by automating image analysis and reducing inter-reader variability. Biomarker-driven methods identify molecular alterations in patients before imaging signs of cancer are evident. Both AI and liquid biopsy show the potential to improve sensitivity and specificity, enabling the detection of early-stage cancers that traditional methods, like low-dose CT (LDCT) scans, might miss. Integrating AI and biomarker-driven methods offers significant promise for transforming lung cancer screening. These technologies could enable earlier, more accurate detection, ultimately improving survival outcomes. AI-driven lung cancer screening can achieve over 90% sensitivity, compared to 70–80% with traditional methods, and can reduce false positives by up to 30%. AI also boosts specificity to 85–90%, with faster processing times (a few minutes vs. 30–60 min for radiologists). However, challenges remain in standardizing these approaches and integrating them into clinical practice. Ongoing research is essential to fully realize their clinical benefits and enhance timely interventions.

## 1. Introduction

Lung cancer is the leading cause of cancer-related mortality worldwide, responsible for approximately 1.8 million deaths annually [1]. Despite significant advances in cancer treatment, the survival rate for lung cancer remains low, with a five-year survival rate of around 20% [1,2]. This poor prognosis is primarily attributed to late-stage diagnosis, as more than 50% of lung cancer cases are diagnosed at a stage where curative treatment options are limited [1,2]. Therefore, early detection of lung cancer is crucial for improving survival rates, yet current screening methods are still far from being applicable in a real-world setting [2,3,4,5,6,7,8,9,10,11,12,13,14,15,16]. Traditionally, lung cancer screening has relied on chest X-rays and low-dose computed tomography (LDCT). Large-scale studies, such as the National Lung Screening Trial (NLST) and the NELSON trial, have demonstrated that annual LDCT screening can reduce lung cancer mortality by 20–24% in high-risk individuals (e.g., those with a significant smoking history). Beyond imaging, researchers are investigating the use of blood-based biomarkers (e.g., circulating tumor DNA) to complement LDCT screening [17,18,19,20]. However, these methods face limitations, including high false positive rates, significant inter-reader variability, and challenges in detecting early-stage malignancies. Other barriers include eligibility criteria, costs, and insurance issues. In response to these challenges, emerging technologies such as artificial intelligence (AI) and biomarker-driven strategies, including liquid biopsy, are showing great promise in enhancing the accuracy, efficiency, and accessibility of lung cancer screening. AI and machine learning are increasingly being used to analyze LDCT images, improving the accuracy of nodule detection and reducing false positives. Recent studies have shown that AI-assisted screening can enhance radiologists’ ability to identify early-stage lung cancers, potentially leading to earlier interventions. This review aims to evaluate the current state of AI and biomarker-based screening methods, examining their potential to improve early detection, reduce unnecessary interventions, and ultimately revolutionize lung cancer screening [20,21,22,23]. Liquid biopsies hold the potential to revolutionize cancer care through the non-invasive early detection of tumors. A key challenge lies in developing robust tests that can analyze high-dimensional data from numerous blood samples across diverse patient groups. Artificial intelligence (AI), with particularly deep generative models, offers promising solutions.

## 2. Methods

### Query Strategy of Reivew of AI and Lung Cancer Screening

This review is registered in the International Prospective Register of Systematic Reviews (PROSPERO ID: CRD42025641097). A comprehensive literature search was conducted to identify studies and reviews, published between 1 January 2010 and 31 January 2025, that focused on AI in lung cancer screening and biomarker-driven approaches. The following databases were queried: PubMed, Cochrane, and Web of Science. The search terms included: “AI in lung cancer screening”, “artificial intelligence in CT imaging”, “biomarker-driven screening for lung cancer”, “liquid biopsy in lung cancer detection”, and “artificial intelligence in PET imaging”. Studies were selected based on their relevance to the topic, the quality of study design, and clinical applicability.

Inclusion criteria consisted of studies that focused on AI algorithms for CT and PET scans, as well as those discussing the role of liquid biopsy in early lung cancer detection. Studies were excluded if they focused on animal models, did not provide relevant data on AI or biomarkers in lung cancer screening, or were solely focused on pulmonary nodule detection without any connection to AI models or lung cancer screening studies. Studies that concentrated only on radiological behavior and AI without any clinical impact were also excluded.

Additionally, we eliminated studies that exhibited classical biases in AI application, such as diverse algorithms, non-representative populations, and variations in the evaluation of certain variables (e.g., sex, ethnicity, and comorbidities) when these factors were not similarly accounted for by the AI software (DeepMind’s Gemini 2.0 Flash).

## 3. Results

**Figure 1:**
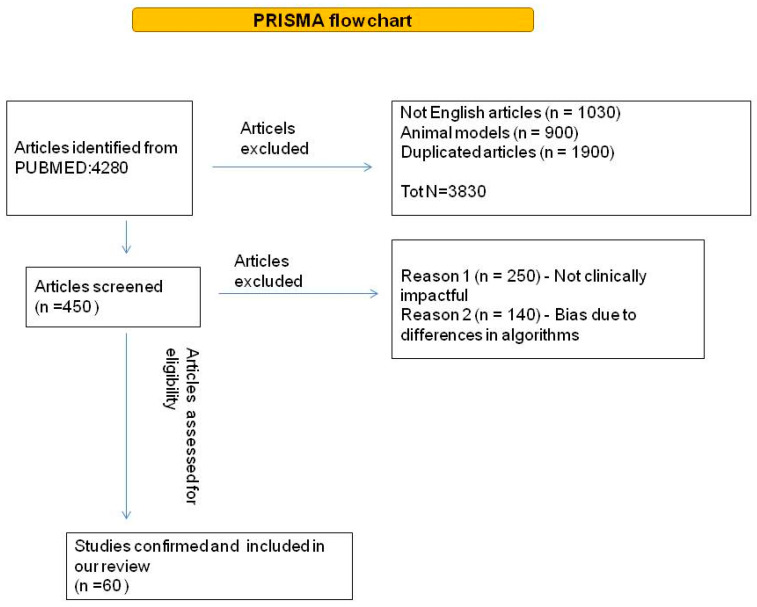
Diagram of literature query flow.

The literature search identified 4280 papers. Figure 1 shows the process of evidence acquisition. Overall, 45 studies were retrieved and fully evaluated. Based on the evidence acquired we discussed: (i) the application of AI in lung cancer screening; (ii) the role of AI in lung cancer imaging and screening; and (iii) biomarker-based screening.

### 3.1. Artificial Intelligence in Lung Cancer Screening

AI (including radiomics, machine learning, and deep learning algorithms) has made significant advancements in improving the sensitivity and specificity of lung cancer detection. These algorithms are especially valuable in analyzing large datasets of medical images, such as CT and PET scans, to automate image analysis and reduce inter-reader variability. A key study by Liu et al. showed the importance of adopting AI in lung cancer screening [9]. In this retrospective study, the authors evaluated 120 CT images and compared the radiologist diagnostic approach with deep learning. They demonstrated that deep learning models could detect small pulmonary nodules on low-dose CT scans with an improvement in sensitivity from 80% to 90%. This 10% increase in sensitivity can result in earlier detection of lung cancer, particularly in cases with smaller or less visible lesions [9]. Additionally, AI algorithms are capable of integrating multi-modal imaging data, combining structural information from CT with functional data from PET scans to assess metabolic activity.

Gao Liang [24] created a radiomics model using monochromatic dual-energy CT (DECT) scans to detect solitary pulmonary nodules, achieving an AUC of 0.8772 (95% CI 0.780–0.974). These results emphasize that CT characteristics can differ among solid pulmonary nodules of various sizes, and identifying size-dependent CT traits can help reduce uncertainty and differentiate benign solid nodules from malignant ones. A more refined distinction of solid pulmonary nodules holds significant clinical importance. Shi et al. [25], evaluating data from more than 2500 nodules, developed another AI-based algorithm. The authors aimed to improve the ability to differentiate between benign and malignant mixed-density ground-glass nodules with a predominant solid component (CTR ≥ 50%), and developed an AI-driven radiomics prediction model (using a Lasso regression), obtaining encouraging results [25].

### 3.2. The Role of Artificial Intelligence in Lung Cancer Imaging and in Lung Cancer Screening

AI’s application in medical imaging has primarily focused on the use of deep learning (DL) for the analysis of CT scans. Deep learning algorithms, such as convolutional neural networks (CNNs), are designed to learn patterns and features within complex data. Studies have shown that CNNs can improve the accuracy of nodule detection and other abnormalities, significantly impacting early diagnosis. Deep learning algorithms are capable of detecting smaller nodules compared to human radiologists, and they are particularly useful in reducing false positives and false negatives. Liu et al. [9] developed a deep learning model for detecting malignant nodules in CT data, achieving higher sensitivity compared to human radiologists [26,27].

Quanyang W et al. [28] showed that with the continuous evolution of AI technologies based on deep learning, particularly the advent of convolutional neural networks (CNNs), AI presents an expanded horizon of applications in lung cancer screening. These applications include lung segmentation, nodule detection, false positive reduction, nodule classification, and prognosis (when we focus on false positives, we always refer to interpretations such as malignancy in a nodule that is not malignant). AI demonstrates significant potential in improving nodule detection sensitivity, reducing false positive rates, and classifying nodules, while also showing value in predicting nodule growth and pathological/genetic typing [26]. AI not only analyzes images but also integrates clinical data to improve risk prediction. AI models can be combined with clinical information, such as smoking history and genetic risk factors, to optimize screening [23,24,25,26,27,28,29]. As a result, AI can identify patients at high risk of developing lung cancer with greater precision compared to traditional methods. AI models need to be trained on large, diverse datasets to ensure they can perform effectively across different clinical contexts and populations. Several clinical studies have evaluated the effectiveness of AI in lung cancer screening for the automated analysis of CT scans, such as in participants of the National Lung Screening Trial (NLST) [3], finding significant improvements in early diagnosis and a reduction in false positives. Additionally, a study by Rajpurkar et al. [30] showed that an AI model trained with millions of CT images achieved sensitivity and specificity comparable to expert radiologists in detecting malignant pulmonary nodules. The authors developed and validated a deep learning algorithm that classified clinically important abnormalities in chest radiographs at a performance level comparable to practicing radiologists [29]. Other studies corroborated these findings [4,11,12,22,30,31,32,33,34,35,36,37,38,39,40,41,42] (Table 1).

### 3.3. Biomarker-Driven Screening: Liquid Biopsy

Biomarker-driven screening, particularly through liquid biopsy, offers a promising complementary approach to imaging-based screening. Biomarker-driven screening has the aim of reducing the false positive rates of conventional imaging-based screening. Liquid biopsy involves the detection of circulating tumor DNA (ctDNA), microRNAs, and other genetic markers in blood samples to identify cancer at early stages, even before tumors are detectable through imaging. This ability to detect molecular alterations before the emergence of visible lesions could significantly enhance early detection and lead to better patient outcomes.

Liang W et al. [28] demonstrated that ctDNA analysis could detect EGFR mutations in 85% of patients with early-stage lung cancer, even before tumors were visible on CT scans. Similarly, Bordi et al. [30] found that liquid biopsy could identify KRAS mutations in 70% of patients with Stage I lung cancer. These findings suggest that liquid biopsy has the potential to detect cancers at stages when curative interventions are still possible.

Recent studies have also suggested that combining liquid biopsy with LDCT screening could enhance diagnostic accuracy. By integrating genetic testing with imaging, the combined approach could help identify high-risk individuals more effectively, reduce unnecessary follow-up procedures, and allow for earlier, more targeted interventions. However, liquid biopsy remains in the validation phase, and more studies are needed to standardize the assays and assess their clinical utility [4,22,29,30,31,32].

## 4. Discussion

AI-driven screening is a generalized term encompassing the application of artificial intelligence techniques in screening processes. This broad category could involve a variety of AI methodologies, ranging from expert systems that rely on predefined rules to more sophisticated machine learning (ML) models. However, it lacks the precision necessary to differentiate between various AI technologies and specify the particular underlying algorithms employed, making it less useful for technical clarity in a scientific context. “Machine learning-based screening” specifically refers to the utilization of machine learning algorithms in the screening process. ML methods involve the development of models that learn patterns and make predictions by analyzing large datasets. This approach distinguishes itself from rule-based AI systems by relying on data-driven techniques, where the model iteratively improves its performance through exposure to training data. Supervised, unsupervised, and reinforcement learning are the primary paradigms within machine learning, and these methods can vary significantly in their approach to learning and pattern recognition. Consequently, machine learning-based screening provides a more structured and algorithmically defined framework than general AI-driven approaches, offering higher accuracy and adaptability in predictive tasks.

“Deep learning models”, a subset of machine learning, utilize multi-layered neural networks, often referred to as deep neural networks (DNNs), to model complex relationships within data. These models are particularly effective in handling large-scale, high-dimensional data, such as images, speech, or textual information. Deep learning relies on hierarchical architectures, where each successive layer extracts increasingly abstract features from the input data. This capability makes deep learning models exceptionally powerful for tasks like image classification, object detection, and natural language understanding. However, deep learning models require significantly more computational resources and vast amounts of labeled training data to achieve optimal performance compared to traditional machine learning models. While deep learning represents a more specialized approach within machine learning, its increased computational demands and complexity make it suitable for highly specific and data-intensive applications. Lung cancer screening, particularly using low-dose computed tomography (LDCT), has proven effective in reducing mortality in high-risk populations. The National Lung Screening Trial (NLST) [26] demonstrated that LDCT could reduce lung cancer mortality by 20% in high-risk individuals. Further studies, such as the NELSON trial, have reinforced these findings, with a 26% reduction in mortality in men and a 61% reduction in women [6,9]. Similarly, the ITALUNG trial showed a 39% reduction in mortality for men and 50% for women [32]. These results underscore the effectiveness of LDCT in reducing lung cancer mortality when applied to high-risk populations [6] (see the details in Table 2). However, despite these advancements, several challenges remain. One of the primary issues is the high false positive rate associated with LDCT, which leads to unnecessary biopsies and follow-up imaging. These procedures are not only costly but also contribute to patient anxiety. Furthermore, overdiagnosis remains a concern, particularly in the case of indolent cancers that would not have caused symptoms during the patient’s lifetime [3]. The published evidence supports the efficacy of LDCT as a screening tool for reducing lung cancer mortality in high-risk populations. In his thorough analysis, Field JK et al. [6] showed that the results align with those of landmark trials such as the NLST and the European Randomized Study of Screening for Lung Cancer (NELSON) [9], both of which demonstrated significant reductions in lung cancer mortality with LDCT. However, the effect size varies, with some trials showing larger benefits than others. The observed heterogeneity may be attributed to differences in study design, participant characteristics, and screening protocols.

Despite its benefits, lung cancer screening with LDCT presents several challenges, including overdiagnosis and false positives. The rate of overdiagnosis remains a significant concern, as some detected cancers may never progress to causing harm. The high rate of false positives leads to unnecessary follow-up tests, biopsies, and patient anxiety [5]. Additionally, the high cost of LDCT and follow-up procedures poses a challenge for large-scale implementation, especially in low- and middle-income countries. Cost-effectiveness analyses suggest that lung cancer screening is only cost-effective in high-risk populations, particularly those aged 55–74 years with a smoking history [7,8,9,10,11,12].

Several studies [4,12,22,32,33,34,35,36,37,38,39] have explored the role of AI in detecting lung cancer, particularly in analyzing CT scans and aiding radiologists in screening and diagnosis. Mehta et al. [40] demonstrated that semi-supervised learning (where AI is trained on partially labeled data) for convolutional neural networks (CNNs) provides a performance comparable to supervised learning, despite using less labeled data. This method enhances AI’s ability to work with more diverse datasets, which is a key advantage [34]. Chamberlin et al. [4] showed that AI’s agreement with expert radiologists in detecting lung cancer on low-dose CT scans was high, with excellent sensitivity (0.929–1) and specificity (0.708–0.960). Chauvin et al. emphasized the role of AI in enhancing the positive predictive value (PPV) during lung cancer screening, thereby reducing false positives and negatives [22]. Cheng et al. [33] compared AI’s performance in detecting lung nodules to radiologists with varying levels of experience. While AI performed similarly to junior radiologists, it was outperformed by more experienced physicians [33]. This suggests that AI can serve as a useful screening tool but still requires expert supervision. Zhang et al. [12] assessed two AI models (CNN and radiomics) and found that while the CNN model had better specificity, radiologists showed higher sensitivity, indicating that AI could match junior radiologists in nodule detection [12]. Studies like those by Armato et al. and Shah et al. have demonstrated that AI can not only detect but also accurately measure tumor size, a crucial step in lung cancer diagnosis [35,36,37]. Yoo et al. found that AI-assisted reading of chest radiographs improved sensitivity for junior radiologists, while senior radiologists saw an improvement in specificity, underscoring AI’s value for both novice and expert clinicians [41]. The AI system tested by Park et al. [36] showed potential in automating Lung-RADS categorization, reducing observer variability. Additionally, Adams et al. demonstrated that AI could enhance Lung-RADS by adding a malignancy risk score (mSI), improving sensitivity and specificity, and reducing diagnostic delays [11,38,39,40,41].

Most AI algorithms, particularly deep learning (DL) models, require large, well-labeled datasets, which are difficult and time-consuming to curate. Strategies like using publicly available databases, generative adversarial networks (GANs), and transfer learning are being explored to overcome this. Challenges remain in dataset quality and consistency, as pathological data can vary in interpretation, and inconsistent terminology is often used to describe nodules. Standardization of labeling practices is needed. Ethical and legal concerns regarding data sharing, privacy, and regulatory compliance are also obstacles, compounded by international data protection laws. Additionally, deep learning models often face overfitting, where performance is good on specific datasets but poor on new data. This can be addressed with larger, more diverse datasets and model validation techniques like cross-validation. The lack of interpretability of AI models remains a concern, but incorporating non-imaging data may help with acceptance. Lastly, rigorous validation is essential to ensure AI algorithms perform well in clinical practice, with careful management of overfitting and underfitting during model training [45].

Liquid biopsies hold the potential to revolutionize cancer care through the non-invasive early detection of tumors. A key challenge lies in developing robust tests that can analyze high-dimensional data from numerous blood samples across diverse patient groups. Artificial intelligence (AI), particularly deep generative models, offers promising solutions. For example, the Orion model can learn generalizable signatures of blood-based biomarkers, such as orphan non-coding RNAs (oncRNAs), by using variational autoencoders. The Orion model uses variational inference to learn a Gaussian distribution from oncRNA data. It surpasses traditional methods in performance and generalizability, achieving high sensitivity and specificity in cancer detection. Orion leverages a two-arm semi-supervised multi-input variational autoencoder to model the expression of oncRNAs and annotated small RNAs, while also incorporating classification and contrastive learning objectives to improve label prediction and remove confounders. This approach enables the model to minimize technical variations and improve the overall accuracy in cancer detection [44,46,47,48,49].

Artificial intelligence (AI) has gained attention for its potential in diagnosing Ground-Glass Opacities (GGOs), with most research focusing on CT images [42,50,51]. However, there is limited research on AI methods for predicting the malignant risk of GGOs using 18F-FDG PET/CT images, particularly in light of the new WHO classification for GGOs [52,53,54]. The 3D nnU-net, an automatic segmentation method based on convolutional neural networks, demonstrates excellent generalization, accuracy, reliability, and efficiency in medical image processing. This study applied the 3D nnU-net with a majority voting method to predict the malignant risk of GGOs using dual-time-point 18F-FDG PET/CT images according to the new WHO classification [55,56,57,58].

The integration of AI into lung cancer screening offers a promising solution to some of these challenges. AI has been shown to reduce inter-reader variability, automate image analysis, and improve the sensitivity and specificity of nodule detection. AI algorithms can also assist in risk stratification, enabling clinicians to identify high-risk patients who may require more intensive monitoring or immediate intervention [11,12,22,33,34,35,36,37,38,39,40,41,42,45]. Biomarker-driven approaches, particularly liquid biopsy, could further enhance screening accuracy by detecting genetic mutations associated with lung cancer at an early stage, before lesions become visible on imaging. Liquid biopsy can complement traditional imaging methods, offering a non-invasive and highly sensitive means of early cancer detection. However, the clinical implementation of liquid biopsy is still in the validation phase, and there are ongoing efforts to standardize testing protocols and establish its utility in routine practice.

## 5. Conclusions

AI and biomarker-driven approaches represent transformative tools for the future of lung cancer screening. AI can enhance diagnostic accuracy by automating image analysis and integrating multi-modal data, while liquid biopsy provides a promising method for detecting genetic alterations at an early stage, even before visible lesions appear, and for reducing the false positive rate. These advancements hold significant potential to improve early detection, reduce unnecessary interventions, and ultimately enhance patient outcomes. AI has the potential to significantly improve healthcare efficiency, especially in regions with limited access to physicians. AI’s introduction in lung cancer screening could lower costs and improve early detection, leading to better patient outcomes. It is anticipated that AI will complement, rather than replace, radiologists in the near future, enhancing clinical decision-making and potentially expanding screening programs worldwide. AI shows great promise in supporting lung cancer screening, particularly in the categorization of findings and reducing the workload of radiologists. While it is unlikely to replace radiologists in the next two decades, AI will become a vital adjunct in clinical decision-making, potentially transforming lung cancer diagnosis and treatment. However, the widespread adoption of these technologies faces several challenges, including the need for rigorous validation, standardization, and addressing disparities in access to care. Further research and collaboration among clinicians, data scientists, and regulatory bodies are essential to overcoming these challenges. If these obstacles can be addressed, AI and biomarker-driven methods could revolutionize lung cancer screening, reducing mortality and improving survival rates globally.

## Figures and Tables

**Table 1:** Published evidence about AI and lung cancer screening.

Study	Objective	Methodology	Data	Key Findings/Results
Armato et al. (2005) [35]	To evaluate an automated lung nodule detection method on low-dose CT scans from a lung cancer screening program.	Automated nodule detection using gray-level thresholding, morphologic modifications, and rule-based/linear discriminant classifiers.	470 nodules from low-dose CT scans.	A 70% sensitivity was achieved with a mean of 1.6 false positives per section. Performance varied based on nodule malignancy, size, subtlety, and radiographic opacity.
Shah et al. (2005) [37]	To investigate computer-aided diagnosis (CAD) in differentiating malignant from benign nodules using volumetric and contrast enhancement features.	Quantitative analysis of nodule size, shape, attenuation, and enhancement from volumetric CT data before and after contrast injection. Classifiers included LDA, QDA and logistic regression.	35 volumetric CT datasets of solitary pulmonary nodules (SPN) with known diagnoses.	Logistic regression classifier on solid ROI features achieved an AZ of 0.926. Addition of GGO features often did not improve performance6. Semi-automated ROI segmentation was a limitation
Zhang B. et al. (2022) [43]	To develop a lung tumor segmentation model using attention mechanisms.	Segmentation squeeze and excitation UNet with multi-scale strategy and dense connected CRF, using different attention mechanisms like SegSE, SE, and CBAM blocks.	759 CT scans (402 NSCLC, 162 LIDC).	The M-SegSEUNet model achieved a Dice coefficient of 0.84211. The model can be extended to other medical image segmentation problems.
Adams et al. (2022) [38]	To evaluate an imaging classifier (mSI) combined with Lung-RADS for lung nodule classification.	Machine learning classifier trained using National Lung Screening Trial (NLST) data, with retrospective testing on external cohorts.	Data from NLST, tertiary referral screening, and non-screening CT datasets.	The mSI, combined with Lung-RADS, showed comparable results to existing clinical risk models and can reclassify nodules15. The mSI may help reduce false positives16.
Shi et al. (2025) [27]	To develop an AI-driven radiomics model to predict the nature of solid-component-predominant pulmonary nodules with CTR ≥ 50%.	Radiomics features extracted from CT images, combined with clinical data; logistic regression to develop a predictive model.	Data from five medical centers, with 370 cases total.	Patient age, volume of solid components, and mean CT value were significant predictive factors. The AUC was 0.721 (training) and 0.757 (validation), indicating moderate accuracy.
Cheng et al. (2022) [33]	To classify peripheral lung cancer (PLC) and focal pneumonia on chest CT images using a 3D CNN with various window settings.	3D CNN trained on segmented lung regions, with orientation alignment and varying window settings.	Retrospective data from 357 patients with PLC or focal pneumonia from chest CT scans.	The neural network achieved an average accuracy of 91.596% in 5-fold cross-validation. Evaluated impact of window settings on AI results.
Park et al. (2021) [42]	To analyze the computer-aided detection (CAD) of subsolid nodules (SSNs) in CT scans.	CAD system applied to CT images to analyze subsolid nodules.	308 patients with SSN and a control group of 182, from a single medical center.	Evaluated the CAD system for detecting subsolid nodules with varying characteristics.
Chauvie et al. (2020) [22]	To compare the performance of different approaches in reducing false positives in a lung cancer screening program.	Comparison of binary visual analysis, lung-RADS, linear regression, machine learning (Random Forest, Neural Network) for nodule classification using Digital Tomosynthesis (DTS).	1594 subjects enrolled in the study.	Neural network was the best predictor with a PPV of 0.95 and a sensitivity of 0.9026. Radiomics features were computed on the central slices.
Zhang Y. et al. (2022) [11]	To evaluate lung nodule detection using AI-assisted reading in actual radiology reports.	AI-assisted analysis using InferRead CT Lung software (V1), compared to radiologist observations.	860 asymptomatic patients who underwent low-dose CT screening.	AI sensitivity for solid and non-solid nodules was 0.988–1, while radiologists had lower sensitivities (0.252 for non-solid and 0.524 for solid).
Zhang R. et al. (2022) [12]	To determine the diagnostic and prognostic value of deep learning/radiomics for solid lung nodules.	3D CNN and Random Forest models were used with clinical and radiomics features.	720 patients with 720 solid lung nodules.	CNN with clinical features had a sensitivity of 0.778, RF with radiomics features had a sensitivity of 0.747, and junior radiologists had a sensitivity of 0.884. Models had higher specificity than radiologists.
Zhang Y. et al. (2023) [44]	To build a high-performance, open-source NER model for LDCT reports and compare it with Stanza model.	Training of rule-based and Bi-LSTM models for named-entity recognition, evaluated against a published open-source model (Stanza).	8305 LDCT reports, with manual annotation for training and testing.	The Bi-LSTM model (F1 score of 0.950) outperformed Stanza (F1 score of 0.872). The model identifies clinically relevant information with high precision and recall.
Chamberlin et al. (2021) [4]	To determine if AI can identify risk factors for cardiopulmonary disease on low-dose chest CT.	AI prototype for detection of lung nodules and coronary artery calcium; per-patient validation against expert radiologists.	Large set of chest CT scans.	The AI prototype rapidly and accurately identifies significant risk factors for cardiopulmonary disease on low dose CT. Age is associated with false positives, and AI may be more sensitive in detection than experts

**Table 2:** Summary of key historical studies on lung cancer screening.

Study Name	Year	Population Description	Key Findings
National Lung Screening Trial (NLST)	2011	U.S. adults aged 55–74 years, heavy smokers or former smokers	LDCT screening reduced lung cancer mortality by 20% vs. chest X-ray
NELSON Trial	2020	European adults aged 50–75 years, at high risk due to smoking history	LDCT screening reduced lung cancer mortality by 26% in men and 61% in women
ITALUNG Trial	2020	Italian adults aged 55–74 years, heavy smokers	LDCT screening reduced lung cancer mortality by 39% in men and 50% in women
Lung Cancer Screening Trial (LUST)	2018	Korean adults aged 55–74 years, former smokers	LDCT reduced lung cancer mortality by 15%
BioMild Study	2021	Italian adults aged 50–75 years, moderate-to-high risk of lung cancer	LDCT screening reduced lung cancer mortality by 39% and increased early-stage detection by 30%
DANTE Study	2023	Italian adults aged 55–74 years, heavy smokers or former smokers	LDCT screening resulted in a 26% reduction in lung cancer mortality and a 40% early detection rate
